# Digestive Symptoms of Pellagra

**Published:** 1911-01

**Authors:** Joseph N. Le Conte

**Affiliations:** Atlanta, Ga.; Lecturer Diseases of the Stomach, Atlanta School of Medicine; Gastro Enterologist to Tabernacle Infirmary and Wesley Hospital


### DIGESTIVE SYMPTOMS OF PELLAGRA.

By Joseph N. Le Conte, M. D., Atlanta, Ga.

*Lecturer Diseases of the Stomach, Atlanta School of Medicine; Gastro Enterologist to Tabernacle Infirmary and Wesley Hospital.*

My experience with pellagra does not cover a large number of cases, but I have tried to notice very carefully the manifestations of digestive disturbance which they have presented, especially as it was on this account that they came into my hands. The first case which came under my observation was referred by Dr. Hutchins in July, 1908, for pronounced digestive disturbance, neither of us at that time recognizing the true character of the disease. He had had her under treatment for several weeks for the eruption on the hands and forearms, which responded promptly to treatment, and at the time I first saw her, was rapidly fading away.

Mrs. R. C. S., 23 years old, been married 5 years. Aunt and grandmother died of consumption. Father has consumption and indigestion.

Patient was never strong. Had measles 3 years ago, and has not been free from indigestion since. Digestive symptoms much worse 8 months ago. Appetite good. Prefers a diet chiefly of bread, butter and syrup. Has pronounced epigastric fullness, and belches much gas having the odor of hydrogen sulphide. Most of the teeth gone, mastication fast and imperfect. Regular stools till two months ago, since then 4 to 12 stools a day accompanied by a great deal of colicky pain in the abdomen. Very nervous. Dizzy for the last 2 months. Headache constant in temples and nape of neck. Dyspnoea. Irregular menstruation for four months. Very weak. Hardly able to walk.

Phys. Exam, 5ft. 5 in. 89 1-2. Anaemic, sallow, marked enteroptosis, 4th degree displacement of the right kidney. Greater curvature of the stomach, 3 finger-breadths below the navel. Cardiac end and lesser curvature also displaced downward. Stomach tender on pressure. Ascending and descending colon palpable and very tender. Other organs normal.

An Ewald test breakfast revealed normal percentages of hcl., pepsin and rennin, and tests for motility showed some muscular insufficiency of the stomach. There was no catarrhal gastritis. The urine was normal, except a very strong indican reaction. A mixed diet stool showed undigested food, quantities of mucus, and the usual finding of a catarrhal colitis.

This patient was under observation for six weeks, improved rapidly in all the symptoms, and at the end of that time moved to N. C., assuring me that she was perfectly well, though examiniation still revealed the presence of enteroptosis and of colitis.

Case 2.—Miss N., 23 years old. School teacher. Family history negative. Had jaundice at three years, had enlarged liver and tympanites at four years, had diphtheria at six, had measles complicated with pneumonia, had continued fever, had rheumatism, had nervous breakdown at High School in 1904, inflammation of the stomach at seminary in 1905. In July, 1909, had so-called typho-malarial fever, in bed three weeks, then began to notice brown spots and cracking on one hand, gradually improved, and began to teach school in Sep., 1909, but obliged to give up very promptly. Has not eaten corn products an average of once a week.

Appetite good. Belches considerable gas after meals. More or less nausea for four months, especially at night. No vomiting, except after Fowler's Sol., in the past two weeks. Great deal of flatulency for two months. Severe griping pains in the bowels at times. Slightly constipated for years till three days ago, when she began to have three to five stools a day. Has seen a great deal of mucus in the stools for the past four months. Has been exceedingly nervous for years, no more now than formerly. Suffers from dyspnoea, occasional palpitation, extreme weakness and tired feeling. Now weighs 103 pounds, at one time 120 pounds.

Phys. Exam. The patient presents the characteristic eruption on the backs of the hands and forearms, though it is now beginning to peel and fade away. Point and edges of the tongue very red, and dorsum of the tongue had lost epithelum in spots,

She is exceedingly weak and reached the office with difficulty on account of nausea and diarrhoea.

Nutrition fair. Habitus normal. Thoracic and abdominal organs normal, except that the epigastrium is tender, the ascending colon is very tender, and the descending colon tender and palpable.

And Ewald test breakfast showed 60 cc. of water soaked crumbs of bread, no free hcl., total acidity 12, phosphates, etc., 10, combined hcl. 2. This and all subsequent examinations showed a pronounced anacid catarrhal gastritis, which still persists. Urine normal, except a very pronounced indican reaction. A mixed diet stool showed the usual findings of a severe catarrhal colitis, with an abundance of mucus.

Patient has been under treatment up to present, with an absence of the months of April and May, spent in N. Y. The diarrhoea disappeared promptly after treatment was begun, all of the symptoms gradually improved, she has gained 18 pounds and says that she feels better than she has in five years.

The gastritis and colitis both persist, though both are greatly improved.

Since we have, as yet, no successful treatment for pellagra, I was obliged, in my cases, to follow the same plan of treatment as if the condition were one of primary digestive disease, which was directed to the betterment of the gastric and intestinal conditions.

None of my cases were able to take arsenic by mouth, owing to its irritating effect on the stomach. Atoxyl hyprodermically was not tried. In several of the cases I tried to use Warburgh's Tincture as a stomachic tonic, but it invariably made the patient worse, and I was obliged to discontinue its use.

In only one of my cases did mental disturbance appear, and that was about two weeks before death.

Several of my cases, while under treatment, would suddenly develop a very sore throat, which would disappear in a few days.

In the cases which have come under my observation, I have been forcibly impressed with the remarkable similarity of the symptoms of the first and second stages of so-called chronic pellagra, to those of chronic catarrhal colitis with or without gastric complications. I believe that, in many cases, it is impossible to make a differential diagnosis between these two conditions, unless the characteristic skin, mouth or mental symptoms are present. Excluding these last characteristic symptoms, the many and varied symptoms presented by one are frequently found in the other in a more or less pronounced form.

The digestive symptoms of pellagra are not at all constant in different cases, (except perhaps the diarrhoea, colicky pain, and gaseous distension), one patient presenting one symptom, and one another. Among those frequently met with are loss of appetite, thirst, heartburn, nausea,vomiting, diarrhoea, sometimes persistent constipation, epigastric pain and tenderness, marked tenderness over a part or the whole of the colon.

The intestinal symptoms are generally manifested before those of the stomach, the latter often not giving serious trouble at all, or only in the terminal stage when persistent nausea, vomiting and inability to retain food may hasten the end.

Any condition of the gastric secretions may be present, the mild cases usually showing normal percentages, and the severe cases or later stages showing marked deficiency. Those having a marked deficiency of hcl. and the ferments usually accompany a catarrhal gastritis, or gastric atrophy, as in case No. 2, recited above.

Dr. Nisbet, of Charlotte, reported at the pellagra congress at Columbia, S. C., last winter, stomach analysis of ten cases, four of which showed marked deficiency of hcl. and five showing excess of mucous. Sixty per cent. of my own cases showed complete absence of hcl. and marked deficiency of the ferments.

Many of the cases show normal motility, while others have defective motility or gastroptosis.

All of my cases showed pronounced catarrh of the colon. The stools seem to be unusually foetid, but I was unable, by careful examination, to find anything constantly present, which does not occur in similar gastro-intestinal disease.

Though we are still at sea as to the positive cause of pellagra, its mode of entrance into the system, and the reason for its attacking certain ones and passing over others in the same environment, the majority of investigators still lean to the theory that it comes from damaged corn, and I have been much interested recently to find a quotation from Neusser, the most noted clinician of Vienna, "that there may be formed in maize a certain mother substance, a non-toxic glucosid, largely under the influence of the BACTERIUM MAYDIS, and that in the intestines this body undergoes decomposition, producing a toxic substance. This decomposition and succeeding toxic formation can occur only when the bowel is in an already diseased condition."

The majority of the reported cases of pellagra which have been acessible to me in the current literature, where a careful history has been taken, relate that the patient had been in impaired health for periods from six months to ten years before the eruption appeared; in many cases the patient described his case as indigestion.

We should remember, in this connection, that chronic catarrhal colitis is a very prevalent and widsepread disease, generally insidious in its onset, and often exists for a year or more before the slowly developing symptoms drive the patient to seek the advice of a physician, and after that drags on for years, without necessarily interfering with the patient's daily vocation.

Moreover, chronic entero-colitis and colitis always become more active in the spring and summer months, mild cases being completely quiescent in the winter only to return in the spring.

My own experience with the disease has been too limited to make any authoritative statement as to the cause, but the cases which I have had the opportunity to study look exactly as if the specific disease pellagra had been engrafted upon a pre-existing entero-colitis.

Whether Neusser's theory is true, that a damaged intestine is necessary for the development of pellagra, I am unable to say, but its seems to be quite possible, and I am positive that even though intestinal inflammation is not a pre-requisite, it frequently paves the way for pellagra by its effect on the vital resistance, and by means of the auto-intoxication which always accompanies chronic catarrhal colitis.

